# Comparative Aspects of BRAF Mutations in Canine Cancers

**DOI:** 10.3390/vetsci2030231

**Published:** 2015-08-24

**Authors:** Hiroyuki Mochizuki, Matthew Breen

**Affiliations:** 1 Department of Molecular Biomedical Sciences, College of Veterinary Medicine, North Carolina State University, Raleigh, NC 27606, USA; E-Mail: hmochiz@ncsu.edu; 2 Center for Comparative Medicine and Translational Research, North Carolina State University, Raleigh, NC 27606, USA; 3 Center for Human Health and the Environment, North Carolina State University, Raleigh, NC 27606, USA; 4 Lineberger Comprehensive Cancer Center, University of North Carolina, Chapel Hill, NC 27514, USA

**Keywords:** dogs, comparative oncology, bladder cancer, urothelial carcinoma, transitional cell carcinoma, mitogen-activated protein kinase

## Abstract

Activating mutations of the *BRAF* gene lead to constitutive activation of the MAPK pathway. The characterization and discovery of *BRAF* mutations in a variety of human cancers has led to the development of specific inhibitors targeting the BRAF/MAPK pathway and dramatically changed clinical outcomes in *BRAF*-mutant melanoma patients. Recent discovery of *BRAF* mutation in canine cancers underscores the importance of MAPK pathway activation as an oncogenic molecular alteration evolutionarily conserved between species. A comparative approach using the domestic dog as a spontaneous cancer model will provide new insights into the dysregulation of BRAF/MAPK pathway in carcinogenesis and facilitate *in vivo* studies to evaluate therapeutic strategies targeting this pathway’s molecules for cancer therapy. The *BRAF* mutation in canine cancers may also represent a molecular marker and therapeutic target in veterinary oncology. This review article summarizes the current knowledge on *BRAF* mutations in human and canine cancers and discusses the potential applications of this abnormality in veterinary oncology.

## 1. BRAF/MAPK Pathway in Cancer Pathogenesis

**Figure 1:**
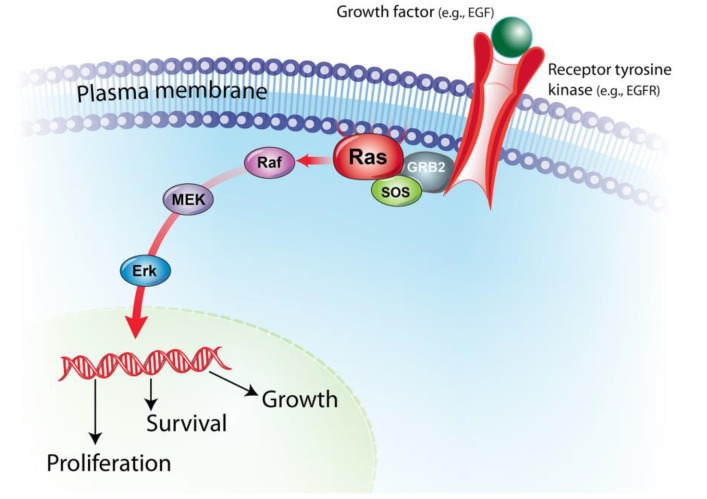
Mitogen-activated protein kinase (MAPK)/extracellular signal-regulated kinase (ERK) pathway. The MAPK/ERK signaling pathway is activated by many different extracellular signals such as binding of growth factors (e.g., EGF) to its receptors (e.g., EGFR). Activated receptor tyrosine kinase phosphorylates and activates RAS family proteins through GRB2-SOS adaptor protein complex. Activated RAS protein, in turn, activates the serine/threonine kinase function of RAF proteins. RAF phosphorylates MEK, which phosphorylates and activates ERK, initiating the signal transduction of many genes involved in various cellular processes.

The mitogen-activated protein kinase (MAPK)/extracellular signal-regulated kinase (ERK) pathway is an evolutionary conserved molecular pathway that regulates fundamental cellular processes, including cell growth, proliferation, differentiation and apoptosis. MAPK pathway signaling is initiated by many different extracellular signals such as growth factors and mitogens. Ligand binding to receptor tyrosine kinases (e.g., EGF and its receptors) triggers phosphorylation and activation of RAS families, which in turn activates RAF proteins. Activation of RAF leads to subsequent activation of MEK, initiating the signal transduction of many genes involved in different cellular processes [1] (Figure 1).

The MAPK pathway is activated in many cancers through different molecular mechanisms, enabling cancer cells to grow independently of extracellular proliferation signals. Somatic mutation of *RAS* genes is one molecular alteration leading to constitutive activation of the MAPK pathway. Activating mutations of three *RAS* genes, *HRAS*, *KRAS* and *NRAS*, are found in 20%–25% of all human cancers [2]. Similarly, *RAS* mutations are present in canine cancers including lung cancer [3], leukemia [4] and other types of cancer [5,6,7].

Activating mutations of *RAF* genes represent another mechanism for constitutive activation of the MAPK pathway. The RAF family consists of three members: ARAF, BRAF and CRAF. Among the three forms of *RAF* genes, *BRAF* gene is most frequently mutated in human cancer [1,8,9]. Since the discovery of *BRAF* mutations in melanoma and other cancers in 2002 [10], a number of studies further identified and characterized *BRAF* mutations in human cancer. The most common (>90%) somatic mutation of the human *BRAF* gene is a T-to-A transversion in exon 15 at nucleotide 1799 (c.1799T > A), resulting in the amino acid substitution from valine to glutamic acid at codon 600 (V600E) [1]. The *BRAF* V600E (*BRAF^V600E^*) mutation occurs within the activation segment of the gene and mimics phosphorylation, drastically elevating kinase activity and activating downstream signal [9,10]. The discovery of *BRAF* mutations and MAPK pathway dependence of human cancers led to the therapeutic strategy targeting BRAF/MAPK pathway.

*BRAF* exon 15 is highly conserved between species; amino acid sequences encoded by exon 15 are identical between humans and dogs, including valine at codon 600 in human *BRAF*. Two recent studies identified canine *BRAF* V595E mutation, a somatic mutation of canine *BRAF* gene orthologous to human V600E, in different types of canine cancers [11,12] (note: this mutation is a T to A transversion at position 8,296,284 on dog chromosome 16 (canFam3.1). Previous studies referred to this mutation as either V595E or V450E, due to the use of different reference sequences. Throughout this paper we use “V595E” to avoid confusion, with a protein sequence based on Ensemble Transcript ID:ENSCAFT 00000006306). Coupled with frequent mutations of *RAS* genes between human and canine cancers, the evolutionarily conserved *BRAF* mutations underscore the importance of MAPK pathway activation as a common oncogenic molecular pathway. This review article summarizes the current knowledge of *BRAF* mutations in human and canine cancers and discusses potential applications of the dysregulation of BRAF/MAPK pathway in veterinary oncology.

## 2. *BRAF* Mutations in Human and Canine Cancers

### 2.1. Melanocytic Tumors

Perhaps the most well described *BRAF*-mutated cancer in humans is melanoma. Melanoma is a cancer originating in melanocytes, occurring mainly in skin (>90%), but also in other locations including eye and mucosal regions [13,14]. Cutaneous melanoma is the fifth and seventh most commonly diagnosed cancer in men and women, respectively, with diagnosis in >70,000 cases and ~10,000 death in the US each year [15]. Furthermore, incidence of cutaneous melanoma has been continuously growing in the Western countries over the past three decades as much as fivefold [16]. Constitutive activation of MAPK signaling plays an important role in the pathogenesis of human melanoma through activating mutations of *BRAF* (~60%) or *NRAS* (~15%) genes [10,17,18,19]. The V600E mutation is the most common form of the *BRAF* mutation in human melanoma [17]. 

Malignant melanoma is the most common neoplasm of the oral cavity in dogs, but also occurs on the skin, digits and eye [20,21]. As in human melanoma, constitutive activation of the MAPK pathway is also implicated in canine melanoma [22,23]. Several studies have examined the presence of *BRAF* mutations in canine melanoma; however, only one study identified *cBRAF^V595E^* mutations in a small percentage of patients (6%) [11,21,22]. The disparity in the prevalence of *BRAF* mutation may result from differences in the role of UV exposure in the pathogenesis of human and canine melanoma. In human melanoma, the presence of *BRAF* mutations is associated with UV exposure, and tumors on mucosal sites or non-UV-exposed skin rarely possess the mutation [24,25]. Unlike humans, the furred-skin of dogs provides natural protection from UV damage. This protection from UV may make dogs less susceptible to UV-related melanoma, resulting in differences in the anatomical location of melanoma between species; the cutaneous form accounts only for ~25% of canine melanoma, with the majority of tumors arising in the oral cavity [21]. The low frequency of *BRAF* mutations among canine melanomas, coupled with UV-independent carcinogenesis and unique anatomical distribution, supports the role of the dog as a spontaneous model for investigation of the BRAF-independent pathogenesis of non-UV-associated melanoma, a rare subtype of human melanoma.

It is noteworthy that benign melanocytic lesions also harbor *BRAF* mutations, in both humans and dogs, with frequencies similar to those of malignant melanoma. The *BRAF* mutation was found in 82% of nevi in humans and in 17% of canine melanocytomas [11,26], suggesting the *BRAF* mutation and consequent MAPK activation may play an important role in the initiation of melanocytic neoplasms, but may be insufficient to cause malignant melanoma without additional molecular alterations.

### 2.2. Urothelial Carcinoma and Prostatic Carcinoma

Urothelial carcinoma (UC), also known as transitional cell carcinoma, is the most common (>90%) form of human bladder cancer. Human UC is subdivided into two distinct entities based on the extent of tumor invasion: non-muscle-invasive bladder cancers (NMIBCs) and muscle invasive bladder cancers (MIBCs). NMIBCs carry a more favorable prognosis with five-year survival of ~90%, while local and distant metastasis is common in MIBCs. MIBCs show more complex genomic alterations including chromosomal aneuploidity, chromothripsis and frequent mutations of the *TP53* gene, reflecting their aggressive biological behavior [27]. Although the *BRAF* mutation is uncommon in human UC [28,29], the MAPK pathway activation through different molecular alterations is implicated in human UC, especially in NMIBCs. Somatic mutations in genes upstream of the MAPK pathway, including *HRAS*, *KRAS* and *FGFR3* genes, were found in ~80% of NMIBCs and ~40% of MIBCs in a mutually exclusive manner, suggesting that mutations of these genes lead to activation of the same pathway [30]. Normal human urothelial cells gain proliferation and survival advantage through *FGFR3* mutations and subsequent MAPK pathway activation [31]. Coupled with the fact that mutations of *FGFR3* gene are more common in NMIBCs, activation of the MAPK pathway may be a fundamental molecular alteration in the initiation of NMIBCs.

Canine UC is the most common malignancy in the lower urinary tract, accounting for ~1%–2% of cancer in this species. Definitive diagnosis of canine UC is made by histological examination of tissue specimens obtained by cystoscopy or surgical biopsy [32]. Although it is often difficult to assess the extent of tumor invasion due to the superficial nature of specimens obtained by cystoscopy, the majority of canine UCs are considered invasive with >90% of tumors invading the bladder wall and 20% having metastasis at the time of diagnosis [33]. Two recent studies identified the *cBRAF^V595E^* mutation in 87% and 67% of canine UC cohorts [11,12]. Similar to the human *BRAF^V600E^* mutation, the *cBRAF^V595E^* results in activation of the MAPK pathway, which can be reversed by a BRAF inhibitor [12]. Given the high incidence of the *BRAF* mutation in canine UC, therapy targeting the BRAF/MAPK pathway may thus offer a novel treatment option for dogs with *BRAF*-mutated UC.

As in the case of UC, *cBRAF^V595E^* was found in 80% of prostatic carcinoma (PC) in dogs [11], but is an uncommon mutation in human PC [34,35]. It is interesting that canine UC and PC share the *BRAF* mutation at similar frequencies. This shared molecular alteration may, however, imply that the majority of canine PCs arise from the urothelium. The cellular origin of “carcinoma of the prostate gland” is controversial in dogs. Several studies demonstrated that canine PC shows highly variable morphological characteristics, some of which resemble UC, complicating histopathological distinction between PC and UC arising from prostatic urethra [36,37]. Immunohistochemical markers also fail to differentiate these two cancers, as canine PC cells express urothelial markers [38,39]. Considering the androgen-independent nature of canine PC, it is now believed that the majority of canine PC originates from prostatic ducts and/or prostatic urethra. The similar frequencies of *cBRAF^V595E^* in canine PC and UC also support this hypothesis. Taken together, the high incidence rates of *cBRAF^V595E^* underscore the importance of BRAF/MAPK pathway in the pathogenesis of UC and PC and may present dog as a suitable model for BRAF/MAPK pathway-targeted therapy for human UC and PC.

### 2.3. Brain Tumors

Meningioma and glioma are two major histological types of primary intracranial malignancies in both humans and dogs. Meningioma is a tumor that arises from meninges, whereas glioma is a broad category of brain tumors originating from glial cells including glioblastoma, astrocytoma, oligodendroglioma and ependymoma. These tumors are further subdivided into each histological subtype [40]. Genetic alteration of the *BRAF* gene and subsequent MAPK pathway activation is a frequent molecular event in a subset of human glioma, astrocytoma. The *BRAF* gene is altered by a missense mutation (mainly V600E) or tandem duplication of *BRAF* locus resulting in the *KIAA1549:BRAF* fusion gene in up to 70% of astrocytoma (especially in picocytic astrocytoma and pleomorphic xanthoastrocytoma), while such alteration is uncommon in other types of glial and non-glial tumors [41,42,43,44]. The identification of BRAF/MAPK dysregulation has promoted the potential use of BRAF inhibitor therapy in neuro-oncology [45,46].

Similar to human brain tumors, *cBRAF^V595E^* has been detected in 15% of canine gliomas, and was undetected in 20 cases of meningioma [11]. Although only 15% of canine gliomas harbored a detectable *cBRAF^V595E^*, it is of note that ~60% of canine gliomas show copy number gain of the gene, which raises the possibility that increase in *BRAF* gene dosage may serve as another mechanism for MAPK pathway dysregulation [47]. The presence of *BRAF* alterations in canine glioma, coupled with anatomical and physiological similarities between canine and human brain tumors, offer further indication that studies of these tumors in dogs may serve as a relevant model to explore the therapy targeting BRAF/MAPK pathway in neuro-oncology.

### 2.4. Hematopoietic Tumors

Mutation of the *BRAF* gene is rare in human common hematopoietic cancers including Non-Hodgkin’s lymphoma [48,49], multiple myeloma [50] and acute and chronic leukemia of lymphoid and myeloid origins [48,51]. Recently, the *BRAF^V600E^* mutation was found in significant proportions of two rare hematopoietic malignancies: Langerhans cell histiocytosis (LCH) and Hairy-cell leukemia (HCL). LCH is a clonal proliferative disease of Langerhans cells, the epidermal antigen-presenting cells. A rare hematologic malignancy, HCL is characterized by expansion of abnormal B cells in bone marrow and spleen. The *BRAF* mutation was detected in 57% of LCH [52] and 100% of HCL [48], all of which were *BRAF^V600E^*. The high prevalence of *BRAF^V600E^* in two human hematopoietic malignancies led researchers to investigate the presence of *BRAF* mutations in canine hematopoietic cancers; however, *BRAF* mutations were not detected in any of 245 canine hematopoietic cancers including tumors of histiocytic (histiocytic sarcoma and histiocytoma), lymphoid (lymphoma, plasmacytoma and acute and chronic lymphocytic leukemia), myeloid (acute myelogenous leukemia) and mast cell (mast cell tumor) origins [11]. This absence of *BRAF* mutations in canine hematopoietic cancers may reflect the fact that dogs do not develop diseases that are the counterpart of human LCH or HCL, or perhaps that the MAPK pathway is activated by different molecular mechanisms such as alterations of *RAS* or receptor tyrosine kinases [4,53,54,55,56].

### 2.5. Thyroid Cancers

The activation of the MAPK pathway, as well as PI3K/AKT pathways, is crucial for the initiation and progression of human thyroid cancers [57]. The *BRAF* mutation has been detected in 45% of papillary thyroid carcinoma (PTC) and in 24% of atypical subtype, whereas the mutation has not been detected in follicular and medullary thyroid carcinoma (FTC and MTC, respectively) [58]. 

Thyroid cancer is the most common endocrine tumor in dogs, with 90% of tumors being malignant [59]. The difference in histopathological distribution of human and canine thyroid cancers exists; the most common histological subtype of thyroid cancer in dogs is FTC, whereas PTC is the most common form in humans [60]. One study examined the presence of *BRAF* mutations in a cohort of canine thyroid cancers comprising 47 FTC and 16 MTC. Although there was no evidence of *BRAF* mutations in the cohort, the same study demonstrated the upregulation of PI3K/AKT pathway molecules in canine thyroid cancers, consistent with human FTC and MTC [5,57]. Absence of *BRAF* mutation and upregulated PI3K/AKT pathway in canine thyroid cancers suggest that activation of PI3K/AKT pathway, rather than MAPK pathway, plays a more important role for the tumorigenesis of FTC and MTC.

### 2.6. Other Cancers

*BRAF* mutation is a common genetic alteration in human epithelial cells including lung and colorectal carcinomas, with frequencies of up to 20%, whereas sarcomas rarely possess the mutation [10,17]. This holds true for canine cancers, where the *BRAF* mutation was detected in pulmonary carcinoma and oral squamous cell carcinoma, but absent in common types of canine sarcoma, including hemangiosarcoma, osteosarcoma and soft tissue sarcoma [11]. The prevalence of the *BRAF* mutation has not been characterized in less common canine epithelial tumors, such as carcinomas of the liver and gastrointestinal tract. In addition, since *BRAF* mutations outside of exon 15 has been poorly investigated in canine malignancies, specific types of canine cancer may harbor *BRAF* mutations in other exons as in the case of human lung carcinoma, where 10%–30% of *BRAF* mutations are located in exon 11 [61,62]. The detection of *BRAF* mutation in canine malignancies is still ongoing, and a characterization of *BRAF* mutations across canine cancers will not only provide further insights into oncogenic roles of *BRAF* alteration in different types of cancers but also lead to a new diagnostic and therapeutic strategy for *BRAF*-mutant canine cancers.

## 3. Clinical Implications of *BRAF* Mutations in Human and Canine Oncology

### 3.1. BRAF Mutation as a Cancer Marker

Detection of *BRAF* mutations has formed the basis of molecular methods of diagnostics and disease monitoring, using DNA isolated from biopsy material, either from surgical biopsy or fine needle aspirates. One example for such attempt to use *BRAF* mutations as a molecular testing is clinical management of thyroid cancer. Thyroid nodules are common lesions found in 4%–7% of the adult population [63]. As most nodules are benign, only 5% are cancerous and require surgical intervention. Cytological examination of nodules is the first step to rule out malignancies. However, inconclusive cytological results often lead to unnecessary diagnostic lobectomy of benign nodules. Patients whose nodules were found to be malignant by diagnostic lobectomy also have to undergo a second surgery for thyroidectomy. To avoid unnecessary surgeries for benign nodules and the two-step surgical management for thyroid cancers, the use of molecular markers has been proposed as an aid for preoperative diagnosis [57]. As nodules with the *BRAF* mutation are ~100% indicative of thyroid cancers (PTCs), *BRAF*-mutated thyroid nodules can be treated by total thyroidectomy without necessitating the diagnostic lobectomy [57,58]. Although the *BRAF* mutation alone is not sensitive enough to detect majority of thyroid cancers, a test to detect other molecular abnormalities in combination with *BRAF* mutations were found to increase the sensitivity [64].

Recent advancement of genome technology makes it possible to now detect low numbers of mutant sequences in blood-derived cell-free DNA as a means of disease monitoring and molecular profiling without biopsy of primary tumor (called “liquid biopsy, reviewed in [65]). The detection and/or quantification of circulating *BRAF* mutant alleles is correlated with presence of metastasis, drug response and clinical outcomes in melanoma patients, suggesting that circulating detection of a *BRAF* mutation can be a non-invasive molecular marker for disease monitoring and treatment selection [66,67].

In veterinary medicine, a major challenge in the clinical management of canine UC and PC lies in early and accurate diagnosis, as these cancers are often diagnosed at advanced stage [32,68]. Currently, reliable diagnostic tests for PC and UC are limited to histopathological examination of a tissue, which involves general anesthesia and expensive procedures such as cystoscopy and a surgical biopsy of bladder or prostate. Although cystoscopy is considered a less invasive and preferable method for UC tissue collection, cystoscopy may not be applicable to all dogs, depending on their size and sex as well as availability of the equipment [69]. In addition, tissues obtained through cystoscopy are generally small and sometimes pose a diagnostic challenge to pathologists, underscoring the necessity of access to a diagnostic means that can detect even a small number of tumor cells. A potentially highly sensitive molecular assay for the detection of malignant epithelial cells in free catch urine has been developed using fluorescence *in situ* hybridization (FISH) to detect numerical chromosomal change in UC tumor cells [70]. Coupled with the FISH analysis, the unique high incidence rates of *BRAF* mutation in canine PC and UC suggest that this mutation may serve as a potential cancer marker for these diagnostically challenging cancers. Since UC and PC cancer cells often shed into urine [32,68,71], detection of the canine *BRAF* mutation in urine offers utility as another non-invasive molecular diagnostics for canine UC and PC.

Although the *BRAF* mutation can be a promising molecular marker for these cancers, detection of the mutation in urine may be challenging due to several reasons. First, it is expected that neoplastic cells represent only a minor fraction of nucleated cells in urine. Secondary cystitis is common in dogs with UC and PC, resulting in dilution of the tumor cell population by inflammatory cells in urine [32,72]. One study demonstrated that the *cBRAF^V595E^* mutation was detectable in urine samples of dogs with UC by next generation sequencing of a targeted PCR amplicon; however, the mutation was not detected in some urine samples when the same samples were analyzed by PCR-RFLP, a less sensitive method for mutation detection, suggesting that employment of a sensitive method is a key for the mutation detection in urine samples [12]. Another technical challenge is the existence of PCR inhibitors in urine-derived DNA. It is well-known that PCR inhibitors are co-purified when isolating DNA from urine, compromising PCR efficiencies at various degrees [73,74]. This is most problematic when detection assays rely on PCR efficiency (e.g., quantitative PCR), as the presence of PCR inhibitors could lead to underestimation of target concentrations or false-negative results, especially for targets of a small number of copies. Molecular techniques more resistant to PCR inhibitors, such as digital PCR [75], may need to be employed for reliable detection of *cBRAF^V595E^* in urine samples. 

### 3.2. BRAF/MAPK-Targeted Therapy

Discovery of *BRAF* mutations in a wide variety of human cancers opened a new era of BRAF/MAPK targeted therapy for *BRAF*-mutant cancers. In particular, efforts have been focused on targeting mutant BRAF for treatment in patients with metastatic melanoma. Prognosis for patients with stage IV disease (tumors with distant metastasis) is dismal with one-year survival rate of 33%–62%, due to limited effective treatment options [76]. After the discovery and characterization of *BRAF* mutations in human melanoma, vemurafenib, the first selective inhibitor for mutant BRAF, was developed and evaluated for a treatment option for patients with *BRAF*-mutated metastatic melanoma. In the phase III clinical trial, objective response rates in metastatic melanoma with *BRAF* mutations were 48% for vemurafenib and 5% for dacarbazine, the gold standard treatment for metastatic melanoma at that time, resulting in a significant difference in survival rates at six months (84% in the vemurafenib group *vs.* 64% in the dacarbazine group) [77]. After demonstrating its efficacy, vemurafenib was approved for the treatment in patients with *BRAF*-mutated melanoma by the US Food and Drug Administration (FDA) in 2011. To date, in addition to vemurafenib, several drugs targeting the BRAF/MAPK pathway have demonstrated promising clinical efficacies for *BRAF*-mutated melanoma [78,79]. The BRAF/MAPK-targeted therapy has also shown therapeutic potentials in other *BRAF*-mutated human malignancies [80,81,82]. 

The presence of orthologous *BRAF* mutation in canine cancers raises the possibility that targeting BRAF/MAPK pathway may also provide therapeutic benefits for canine cancers with *cBRAF^V595E^*, especially canine UC and PC. The presence of a *BRAF* mutation, however, does not always correlate with clinical response for BRAF inhibitor. In human non-melanoma malignancies, the therapeutic efficacy of BRAF inhibitors is highly variable between different types of cancer, despite the presence of the *BRAF^V600E^* mutation [83]. *BRAF*-mutant cancer cells can negate antitumor effects of BRAF inhibition by activating different signaling pathways for cell survival and proliferation. For example, unlike melanoma, BRAF inhibitor monotherapy showed limited clinical response for patients with colorectal adenocarcinoma (CRC) harboring *BRAF^V600E^* [84]. This de novo resistance is caused by reactivation of EGFR signaling in CRC cells by disrupting a negative feedback loop that suppress EGFR signaling [85]. The re-activated EGFR signaling allows CRC cancer cells to proliferate in the presence of BRAF inhibition. A similar bypass mechanism of BRAF inhibition is also reported in thyroid cancer cells, through reactivation of HER2/HER3 signaling [86]. These human cancer studies showed that *BRAF* mutational status alone does not predict the therapeutic potential of BRAF inhibition for cancers, suggesting that understanding of molecular networks important for cancer cells is crucial. Although a BRAF inhibitor showed anti-proliferative effects for canine UC cancer cells with *cBRAF^V595E^* at relatively high dosage [12], further characterization of *in vitro* and *in vivo* effects of BRAF inhibition are warranted for the clinical application of the BRAF/MAPK-targeted therapy for *cBRAF^V595E^*-mutated canine cancers. 

## 4. Conclusions

Recent discovery of the *BRAF* mutation *cBRAF^V595E^* (orthologous to the human *BRAF* V600E mutation) in a variety of canine cancers underscores the importance of MAPK pathway activation in carcinogenesis. Using the dog as a relevant spontaneous cancer model, this evolutionarily-conserved molecular alteration may provide a unique opportunity to better understand the oncogenic role of MAPK pathway activation and test molecular-targeted therapies. From a veterinary oncology perspective, high frequencies of the *BRAF* mutation in canine UC and PC may represent a promising molecular diagnostic marker and therapeutic target for these clinically challenging cancers. Further studies to characterize the *BRAF* mutation and MAPK pathway dysregulation in canine cancer will benefit both human and veterinary oncology.
